# The Effect of COVID-19 on Pediatric Traumatic Orthopaedic Injuries: A Database Study

**DOI:** 10.5435/JAAOSGlobal-D-22-00012

**Published:** 2022-02-11

**Authors:** Ajith Malige, Alexa Deemer, Andrew D. Sobel

**Affiliations:** From the St. Luke's University Hospital (Dr. Malige and Dr. Sobel), and the Lewis Katz School of Medicine at Temple University (Ms. Deemer).

## Abstract

Stay-at-home orders and other social distancing restrictions had a profound effect on the lives of children during the pandemic. This study characterizes pediatric orthopaedic injuries and in-hospital outcomes during the COVID-19 pandemic and compares them with pre-COVID patterns.

**Methods:**

A retrospective review of pediatric patients presenting to hospitals with Pennsylvania Trauma Systems Foundation designations was performed. All patients younger than 18 years who presented with orthopaedic injuries were included. Patient demographics, injuries, hospital stays, and mortality were compared between the COVID and pre-COVID cohorts.

**Results:**

Overall, 1112 patients were included. During the pandemic, more injuries occurred at home (44.7% versus 54.9%, *P* = 0.01) and fewer at sporting areas, parks, and pools (7.8% versus 1.6%, *P* < 0.01) as well as at schools (3.4% versus 0.5%, *P* = 0.03). Injuries caused by child abuse were more prevalent during the pandemic (5.6% versus 11.0%, *P* < 0.01). Finally, the COVID cohort had a longer mean hospital length of stay (3.1 versus 2.4 days, *P* = 0.01), higher mean number of ICU days (1.0 versus 0.7 days, *P* = 0.02), and higher mortality rate (3.8% versus 1.3%, *P* = 0.02).

**Discussion:**

Pediatric patients sustained injuries in differing patterns during the pandemic, but these led to worse hospital outcomes, including higher mortality rates.

The COVID-19 pandemic has placed continued stress on patients and hospital systems across the United States. The increased time and economic burden caused by the increasing number of patients with severe acute respiratory syndrome coronavirus 2 has highlighted the need for more resources and better action plans.^1^ Early in the pandemic, state and local governments placed stay-at-home orders and other social distancing restrictions, all aimed at curbing the spread of the virus.^2,3^ Although this had a profound effect on the lives of the entire population, children, in particular, were forced to alter their lives drastically. Schools were closed, and extracurricular events including organized sports were halted, forcing children to spend more time at home away from these locations where injuries in children are commonly sustained.^4-6^

Given the shift away from congregant settings, injury patterns in the pediatric population could have been greatly affected. Studies are starting to detail changes in pediatric orthopaedic injury patterns during the pandemic, but all previous studies have been conducted in single center or international settings or did not describe details related to hospitalizations.^7-9^ Bram et al conducted a comprehensive study of pediatric orthopaedic injuries during the COVID pandemic by analyzing patients presenting to sites within one pediatric hospital network. Although their analysis primarily focused on fractures and included patients treated through ambulatory settings, they showed a considerable reduction in fractures and highlighted public health strategies. It is unknown whether these changes have also occurred in a wider variety of communities outside of an urban setting or in pediatric orthopaedic patients with injuries other than fractures. Furthermore, the characteristics of patients hospitalized with orthopaedic injuries during this pandemic have not yet been elucidated. Understanding these patterns will allow for the targeting of resources and public health initiatives during the remainder of this pandemic and future similar situations. This may allow better focusing and utilization of the proposed principles of care during the COVID-19 pandemic.^10-12^

The primary purpose of this study was to compare the location and mechanism of orthopaedic injuries during the COVID-19 pandemic compared with previous years across a diverse geographic spread. The primary hypothesis was that there was a shift away from injuries at school and sporting events toward more injuries at home. The secondary purposes of this study were to compare the prevalence of orthopaedic injuries and procedures and hospital outcomes between patients during the COVID-19 pandemic and previous years. The secondary hypotheses were that there will be a shift toward fewer higher-energy, open injuries during the pandemic without a shift in location of injuries. In addition, there will also be a shift toward worse hospital outcomes and longer lengths of stay during the pandemic.

## Methods

### Study Design

This study was approved by our hospital's institutional review board. The authors requested data from the Pennsylvania Trauma Systems Foundation Trauma Registry (PTSF) for the month of April 2020 (COVID cohort), which was used as a marker for the initial peak of COVID-19 in the United States during which the greatest number of stay-at-home orders were in place. Data from the months of April 2017, April 2018, and April 2019, denoted as the pre-COVID cohort, were also obtained for all traumatic injuries. A retrospective review of all recorded injuries from the 42 included hospitals (40 hospitals in 2017, 41 in 2018 and 2019, and 42 in 2020) with PTSF designations was performed. This included three pediatric trauma centers, three trauma centers with dual adult and pediatric accreditation, and all centers with adult accreditation. All centers have 42 days after a patient is discharged to submit patient data to the registry and must submit at least 85% of their cases to maintain PTSF designation. Patients aged 17 years and younger who had documented traumatic orthopaedic injuries were included. Patients aged 18 years and older, those who did not have documented orthopaedic injuries, and those with incomplete recorded data that did not allow proper classification of a patient into the appropriate cohort were excluded.

All injuries and procedures were represented by *ICD-9* and *ICD-10* diagnosis and procedure codes. All procedures, except the placement of peripheral intravenous lines or dressing changes, regardless of if they were performed in the operating room or at the bedside, were included. Two of the authors coded each diagnosis and procedure by anatomic location and type (bony and soft tissue). Bony injuries were used to define fractures and dislocations, whereas soft-tissue injuries were used to define nerve, vascular, ligament, tendon, muscle, and skin injuries. All periarticular injuries were sorted into their respective joint (eg, distal humerus, proximal radius, and proximal ulna fractures were coded as bony elbow injuries). A reliability analysis using 500 random codes between the two authors yielded an intraclass correlation coefficient of 0.96, with a rating of 0.90 noting an excellent reliability between the two coders.

### Study Outcomes and Statistical Analysis

For each patient, demographic information, injury details, mechanism of injury, hospital stay details, procedures performed, hospital disposition, and mortality were recorded. Injuries occurring as a result of suicide attempts, abuse, or assault were also recorded. Injuries between the cohorts were compared to see whether there was any difference in injury patterns and outcomes during the COVID-19 pandemic compared with the previous years. Demographic information, injury location, and injury mechanism were analyzed using chi-squared tests. Injury anatomic locations and procedures were analyzed using chi-square and Fisher exact tests (if absolute count was less than 5), whereas Mann-Whitney *U* testing was used for alcohol levels and Glasgow Coma Scale levels. Finally, hospital outcomes were analyzed using Mann-Whitney *U* tests for continuous variables (ie, length of stay, intensive care unit days, ventilator days, and complication rate) and the chi-square test for mortality (IBM SPSS Version 23 Statistics for Windows: IBM). For all analyses, *P* < 0.05 denoted statistical significance (Table 1).

**Table 1:** Demographic Information of Our Sample Population

	Pre-COVID	COVID	*P*
Age			
0-4 years	323 (34.7%)	62 (34.1%)	0.85
5-9 years	232 (24.9%)	41 (22.5%)	
10-14 years	212 (22.8%)	46 (25.3%)	
15+ years	163 (17.5%)	33 (18.1%)	
Sex			
Male	585 (62.9%)	119 (65.4%)	0.52
Female	345 (37.1%)	63 (34.6%)	
Race			
Caucasian	599 (64.4%)	114 (62.6%)	0.90
Black	189 (20.3%)	39 (21.4%)	
Other/unknown	142 (15.3%)	29 (15.9%)	
Ethnicity			
Hispanic/Latino	95 (10.2%)	25 (13.7%)	0.37
Non-Hispanic/Latino	810 (87.1%)	152 (83.5%)	
Unknown	25 (2.7%)	5 (2.7%)	
Insurance			
Medicare/Medicaid	475 (51.1%)	98 (%)	0.12
Private Insurance	404 (43.4%)	67 (36.8%)	
Self-Pay	47 (5.1%)	15 (8.2%)	
Unknown	4 (0.4%)	2 (1.1%)	
Total	930 (83.6%)	182 (16.4%)	

Statistical analysis performed using chi-square tests.

## Results

### Demographics

**Figure 1:**
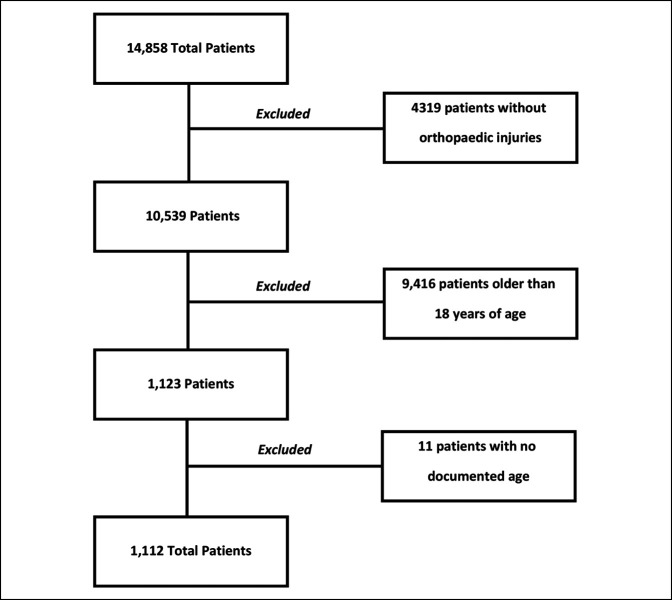
Flowchart showing inclusion and exclusion criteria leading to final cohort

Overall, data from 14,858 patients were reviewed, with 1112 patients included in our study (Figure 1). The pre-COVID cohort (April 2017, April 2018, and April 2019) included 930 patients (monthly mean 310 patients) and the COVID cohort (April 2020) included 182 patients. This represented a 41% reduction in pediatric patients with orthopaedic injuries during the pandemic. Two (1.1%) of the 182 patients in April 2020 tested positive for the SARS-CoV2 virus. The largest demographic groups in our sample were patients who were younger than 5 years (n = 385, 34.6%), male (n = 704, 63.3%), Caucasian (n = 713, 64.1%), Non-Hispanic or Latino (n = 962, 86.5%), nonobese (body mass index of less than or equal to 24.9 kg/m^2^ [n = 121, 1.3%]), and had Medicare or Medicaid insurance (n = 573, 51.5%). There was no difference in demographic breakdown between the two cohorts (Table 1).

### Injury Location and Mechanism

**Figure 2:**
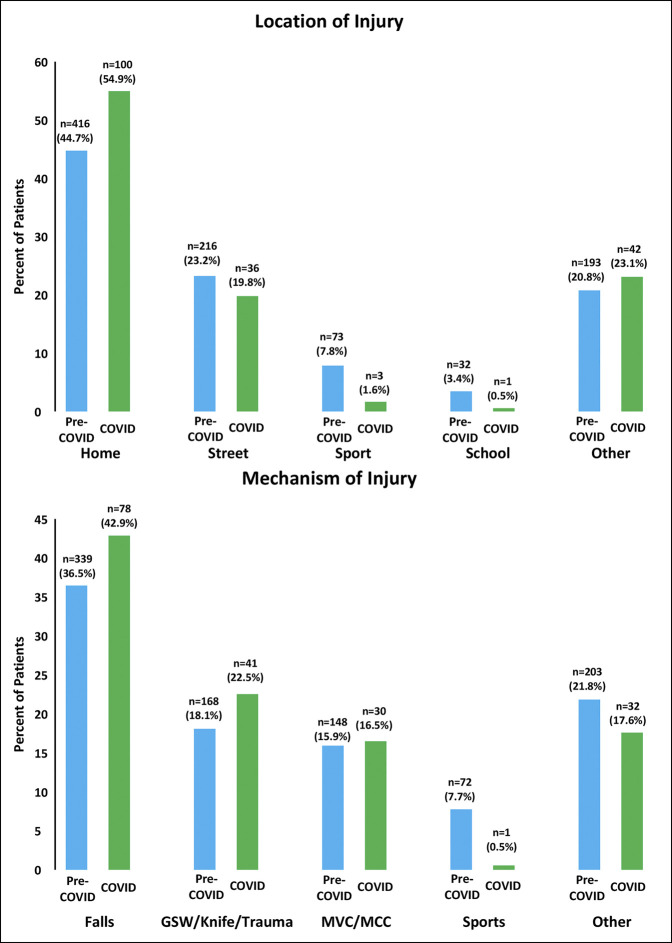
Bar graphs showing the location of injury (**A**) and mechanism of injury (**B**). Statistical analysis of injury location and injury mechanism between the COVID (April 2020) and pre-COVID (April 2017, April 2018, and April 2019) cohorts using chi-squared tests showed a significant difference in location patterns (*P* < 0.01) and injury mechanism (*P* = 0.02) over the four months. Home = injury at home; Street = injury on street, sidewalk, or parking lot; Sport = injury at sporting event, playground, or swimming pool; School = injury at school; Other = injury at other location or unknown location; Falls = falls at ground level or from height; GSW/Knife/Trauma = gunshot wound, knife, child abuse, trauma by another person, or trauma by another object; MVC/MCC = motor vehicle or motorcycle crash; Sports = injury from playing in sporting event or playground.

Most injuries occurred at home in both the pre-COVID (n = 416, 44.7%) and COVID (n = 100, 54.9%) cohorts, although these proportions were different (*P* < 0.01) (Figure 2). A smaller proportion of injuries occurred at sporting areas, parks, and pools (7.8% versus 1.6%, *P* < 0.01) as well as at schools (3.4% versus 0.5%, *P* = 0.03) during the COVID pandemic.

Falls were the most common mechanism in both the pre-COVID (n = 339, 36.5%) and COVID (n = 78, 42.9%) cohorts (Figure 2). There was an overall difference in injury mechanisms between the pre-COVID and COVID cohorts (*P* < 0.02), with children in the COVID cohort being less likely to sustain injuries from playing in a playground or at sporting events (7.7% versus 0.5%, *P* < 0.01).

Overall, there were four reported suicides, 68 reported assaults, and 72 reported abuse cases in our cohort. Although there was no difference between cohorts in assault rate (*P* = 0.97), there was a higher proportion of cases related to abuse during April 2020 compared with the pre-COVID cohort (n = 20, 11.0% versus n = 52, 5.6%; *P* < 0.01).

### Injuries and Procedures

**Figure 3:**
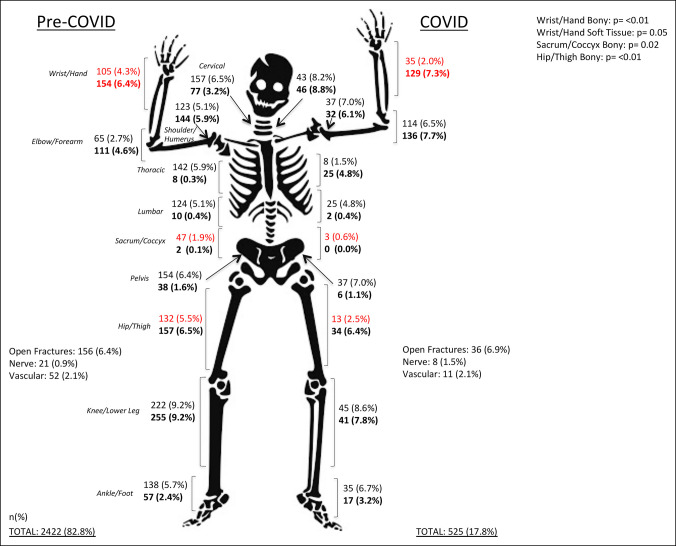
Illustration showing pediatric orthopaedic injury breakdown by type and cohort. All injury anatomic locations and types presented in absolute counts and percent of total injuries. Red values are significant. All bold values represent soft-tissue injuries in the corresponding anatomic location, and all nonbolded values represent bony injuries.

There were 2947 orthopaedic injuries in our study. The pre-COVID cohort sustained 2422 (monthly mean n = 807) injuries, whereas the COVID cohort sustained 525 injuries. This represented a 35% reduction during the pandemic and averaged out to 2.6 injuries per patient in the pre-COVID cohort and 2.9 injuries per patient in the COVID cohort. In the entire sample, there were 649 (58.4%) patients who were polytraumatized with injuries to multiple systems and 698 (62.8%) patients who sustained multiple orthopaedic injuries. Overall, there were 358 (12.1%) burn injuries, 192 (6.5%) open fractures (6 were Type III), 29 (1.0%) nerve injuries, and 63 (2.1%) vascular injuries in the entire sample. Our sample also sustained 1717 (58.3%) bony injuries and 1230 (41.7%) soft-tissue injuries. Patients in the COVID-19 cohort were more likely to sustain injuries to the bony wrist and hand (*P* < 0.01) and soft-tissue wrist and hand (*P* = 0.05) structures, whereas they were also less likely to sustain injuries related to the bony sacrum and coccyx and bony hip and femur (*P* < 0.01) (Figure 3).

**Figure 4:**
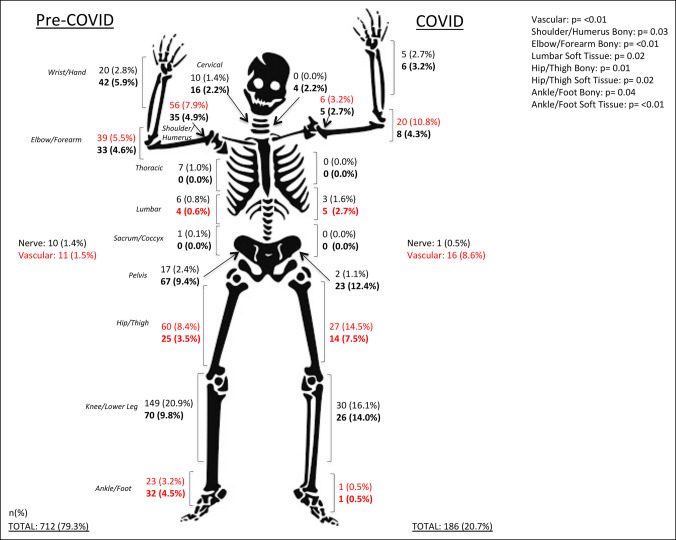
Illustration showing pediatric orthopaedic procedure breakdown by type and cohort. All procedure anatomic locations and types presented in absolute counts and percent of total procedures. Red values are statistically significant. All bold values represent soft-tissue procedures in the corresponding anatomic location, and all nonbolded values represent bony injuries.

Overall, 898 orthopaedic procedures were performed in this study. Seven hundred twelve procedures were performed in the pre-COVID cohort (monthly mean n = 237) and 186 in the COVID cohort, representing a reduction of 22% of orthopaedic procedures performed during the pandemic. Although there were increased proportions of vascular (*P* < 0.01), soft-tissue lumbar (*P* = 0.02), shoulder and humerus bony (*P* = 0.03), elbow and forearm bony (*P* < 0.01), and hip and thigh bony (*P* = 0.01) and soft-tissue (*P* = 0.02) procedures during the pandemic, there was also a lower proportion of foot and ankle bony (*P* = 0.04) and soft-tissue (*P* < 0.01) procedures in the COVID-19 cohort. There was no difference in the number of patients who underwent orthopaedic surgical intervention between the non-COVID (n = 308, 33.1%) and COVID (n = 73, 23.5%) cohorts (*P* = 0.07) (Figure 4). However, each patient underwent an average of 0.76 or 1.02 procedures during the pre-COVID and COVID cohorts, respectively.

**Figure 5:**
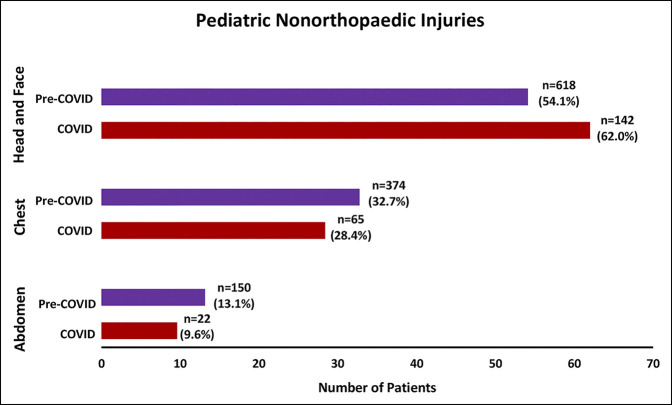
Graph showing pediatric nonorthopaedic injuries. All numbers include total bony and soft-tissue injuries. All percentages presented of total injuries in corresponding location in the cohort. Statistical analysis using chi-square tests comparing the COVID (April 2020) and pre-COVID (April 2017, April 2018, and April 2019) cohorts. All percentages are calculated based on total nonorthopaedic injuries in the cohort.

The total patient sample sustained a total of 1371 nonorthopaedic injuries (Figure 5). No difference in the proportion of patients having concomitant nonorthopaedic injuries was seen between the COVID (n = 110, 60.4%) and pre-COVID cohorts (n = 537, 57.7%) (*P* = 0.50). There was also no difference between cohorts in the proportion of patients who underwent nonorthopaedic procedures (n = 40, 22.0% versus n = 190, 20.4%, *P* = 0.64). Finally, there were 184 other undetailed injuries and 10 undetailed procedures.

Although there was no difference in mean patient blood alcohol levels at admission (n = 172 patients, 0.006 versus 0.007, *P* = 0.66), there were a higher number of patients with a positive drug screen on admission in the COVID-19 cohort (13.2%) compared with the pre-COVID cohort (5.6%) (*P* < 0.01). Finally, there was no difference in the Glasgow Coma Scale score (13.8 versus 13.2, *P* = 0.07) or Injury Severity Score (7.5 versus 9.1, *P* = 0.06) between the COVID and pre-COVID cohorts.

### Hospital Outcomes

**Figure 6:**
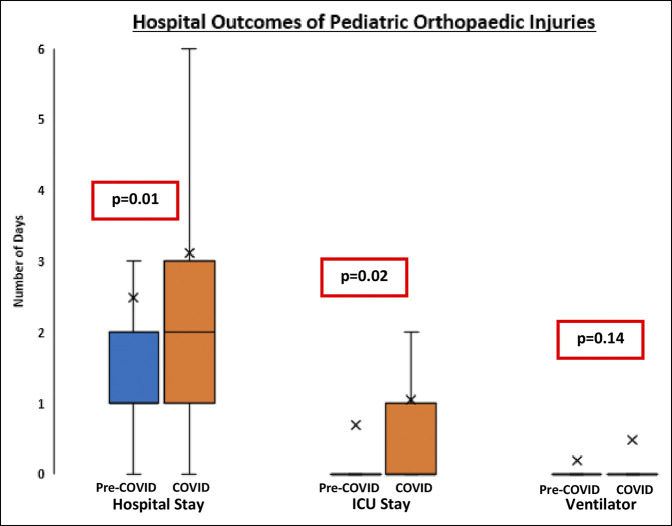
Box plot showing hospital outcomes in pediatric orthopaedic injuries. Mann-Whitney *U* analysis yielded a statistically higher number of hospital length of stay in days (*P* = 0.01) and days spent in the ICU in April 2020 (*P* = 0.02) with no statistical difference between the COVID (April 2020) and pre-COVID (April 2017, April 2018, and April 2019) cohorts in days with ventilator needs (*P* = 0.14). ICU = intensive care unit.

The COVID cohort had a longer mean hospital length of stay compared with the pre-COVID cohort (3.1 versus 2.4 days, *P* = 0.01). The COVID cohort also had a higher mean number of days spent in the ICU (1.0 versus 0.7 days, *P* = 0.02). However, the COVID and pre-COVID cohorts showed no difference in the number of days with ventilator needs (0.5 versus 0.6 days, *P* = 0.14) (Figure 6). Overall, there were 24 reported in-hospital complications, the most common of which were respiratory complications (11 patients) and blood clots, including deep vein thrombosis or pulmonary embolism (five patients). There was no difference in the percentage of patients of complications in the pre-COVID and COVID cohorts (2.0% versus 1.6%, *P* = 0.56). Patients were discharged home in 73.6% of cases in the COVID-19 cohort (n = 134) and 71.5% in the pre-COVID cohort (n = 665). Finally, a higher percentage of patients with orthopaedic injuries died in the hospital during the initial height of the COVID pandemic (n = 7, 3.8%) compared with the matched months before the pandemic (n = 12, 1.3%) (*P* = 0.02). This represents a 75% increase in the number of deaths in the pediatric population presenting with orthopaedic injuries to trauma centers in Pennsylvania during the initial peak of COVID compared with reciprocal months before the pandemic (odds ratio 2.98 [95% CI:1.1579 to 7.6736]).

## Discussion

Government-induced social distancing requirements and lockdowns have altered the lives of many during the COVID-19 pandemic. Children, in particular, had to adapt to life without in-person schooling, sporting events, and other group activities for long periods of time. In an effort to slow the spread of the virus, the government stopped or closed down some of the most common locations at which orthopaedic injuries were sustained in the pediatric population,^4-6^ which this study has shown has changed injury mechanisms, injury types, and distribution of types of orthopaedic procedures performed. We have also seen rises in the numbers of pediatric patients who were victims of abuse, presented with positive drug screens, and died in the hospital. These findings are more generalizable and give more understanding to the effects of the COVID pandemic than previous studies that have focused on single hospitals given the variety of geographic locations, hospital sizes, and patient populations included in our data set.

There was a 41% reduction in pediatric patients sustaining orthopaedic injuries in April 2020 compared with matched months before the pandemic. This is a finding echoed by other studies detailing pediatric orthopaedic injuries during this time,^8,9,13^ which supports the validity of our data set and also our comparative analysis of cohorts. Furthermore, there was no difference in the demographics of these patients sustaining injuries over time, suggesting that the populations of children throughout the state were affected equally by the restrictions.

With social distancing guidelines in effect, people were asked to spend more time at home and less time interacting with others. This shift in behavior and the shutdown of schools and organized sport activities led to an expected increase in injuries at home and decrease at sporting areas, parks, pools, and school. This was accompanied by a decrease in injuries caused by playground accidents or sporting event accidents. These results echo the trends found by Raitio et al. who found a similar decrease in daycare, school, and organized sports-related injuries during the pandemic.^8^

There were multiple disturbing findings within our study of injured children. We noted increases in patients who were victims of child abuse and/or who had positive drug screens. Unfortunately, multiple other studies detailed the rise in cases of child abuse during the pandemic,^14-16^ However, the increased proportion of patients sustaining orthopaedic injuries as a result of abuse noted in the present study is striking. The increasing mental and emotional toll of social distancing, job loss, and time spent at home has been linked to the increased rate of child neglect and abuse.^17^ Even more troubling is the notion that these increases could be even higher due to underreporting of child abuse during the pandemic.^18,19^ These findings have led to an increased push to improve awareness of this problem for families and healthcare professionals and strategies aimed at decreasing the rate of abuse.^20,21^ That orthopaedic injuries are more frequently associated with child abuse during this pandemic should be an incredibly important focal point to address now and before future pandemics or similar situations. Furthermore, an increase in positive drug screens among pediatric patients suggests that access to these substances during the pandemic was heightened. One could surmise that with children spending more time at home and with injuries more frequently sustained at home, that access would come from substances already present at home and targeted efforts for drug safety could improve this statistic.

Pediatric patients sustained fewer bony and soft-tissue injuries during the pandemic compared with before the pandemic. Despite the lower number of injuries as a whole during the pandemic, a greater proportion of injuries sustained were to the bony wrist and hand. In fact, there were more bony wrist and hand injuries during the COVID cohort (48) than the monthly mean pre-COVID (35), which suggests a difference from established literature. Bram et al showed a 50% reduction in patients presenting with distal radius fractures during the COVID cohort.^9^ The difference in our findings can potentially be explained by the inclusion of patients presenting to ambulatory sites in the study by Bram et al and also the consolidation of wrist and hand fractures in our data rather than just distal radius fractures alone.^6^ However, our noted increases in upper extremity orthopaedic injuries during the pandemic could be due to increased participation in activities at home that led to upper extremity blunt force trauma or falls on the upper extremity compared with previous years. Overall, our decrease in overall orthopaedic injuries and procedures performed is consistent with previous reports on pediatric injuries during the pandemic.^7-9,13^

We also noted a decrease in the number of orthopaedic procedures performed during the pandemic. However, we did not see a difference in the proportion of patients with orthopaedic injuries undergoing surgical procedures. With the reductions or shutdown of elective cases in many hospital systems during April 2020, one could reason that an expansion of the availability of operating rooms, anesthesia team members, and resources might allow for an increase in the proportion of patients taken to the OR for surgical management of their injury.^22^ This was not seen and is likely due to the appropriation of the aforementioned to treating patients with COVID or rotation of people and resources out of the hospital settings to protect them from exposure.^23^

Patients who sustained orthopaedic injuries during COVID-19 had worse hospital outcomes compared with the pre-COVID cohort, as detailed by the longer hospital length of stay, ICU stay, and higher mortality. This points to a worse prognosis in patients who presented during the pandemic. Although there have been studies linking certain orthopaedic injuries in patients with COVID-19 with worse outcomes,^2,24-27^ our data set cannot directly link these outcomes to a positive test due to the low number of reported positive tests. Furthermore, these studies have detailed a difference in adult and geriatric populations, not pediatric patients. Instead, the disparity noted in our study likely can be linked to overtaxed physicians and hospital systems with the allocation of resources away from patients with traumatic injuries. It is possible that the limited resources, time, and care available to each patient lead to an unfortunate trend of worse outcomes for patients who presented during this month. This is also seen in the adult population, as all demographics were negatively affected by the overtaxed healthcare system.^28^ Previous studies have also linked the coronavirus to increased mortality rates in the general population.^29^ The prevalence of pediatric patients with undetected (untests or false negative) was substantial during April 2020, and the presence of the virus itself may have had a direct effect on patient outcomes. However, the burden of illness in pediatric patients caused by COVID-19 is much lower than that in adults and certainly in the geriatric population, so it is possible that the effects of the virus were negligible in affecting outcomes.^30^ Finally, children were three times more likely to pass away during the COVID cohort compared with the pre-COVID cohort, although the small absolute number of deaths in our study makes further analysis hard. However, previous studies have understandably shown that poorer and non-White demographics have been at a higher risk for increased mortality rates during this pandemic,^31-33^ a finding that highlights the importance of expanding sufficient health care to all patient populations.

This study has limitations inherent to all database studies. The accuracy and completeness of these data may not be perfect and relies on different staff with differing levels of experience at participating trauma centers. Despite this, centers are mandated to submit data for at least 85% of their patients so the potentially small amount of missing data would be unlikely to contribute heavily to changing the trends seen here. Furthermore, this database is biased toward injuries that require admissions and hospital stays, as there was a relatively small number of patients who were discharged from the hospital the same day that they presented for evaluation It also does not include patients presenting to urgent cares, ambulatory centers, or nontrauma center hospitals, so potentially lower acuity patients are not included in the analysis. Patients who remained admitted to the hospital at the time of data acquisition from this database (6 months after April 2020) were not able to be included in the database, and these individuals who would have potentially needed advanced care, more numerous procedures, longer stays, or who had different hospital outcomes could alter the results. The low number of reported positive COVID tests also does not allow the analysis of the effect of a positive test on patient outcomes. This number may also be lower than the actual proportion of COVID-positive cases within the sample because asymptomatic cases may not have been tested and mandated testing may not have been in place at each trauma center. Finally, although the authors chose to focus on April 2020 as a marker of the height of COVID-19 in our state, there might be differences in injury patterns in other months or other locations. Future studies should look at the entire pandemic across the country to understand the complete picture of any change in pediatric orthopaedic injury patterns due to the pandemic.
